# Comprehensive Overview of Vaccination during Pregnancy in Europe

**DOI:** 10.3390/jpm11111196

**Published:** 2021-11-13

**Authors:** Anca Angela Simionescu, Anca Streinu-Cercel, Florin-Dan Popescu, Ana Maria Alexandra Stanescu, Mariana Vieru, Bianca Mihaela Danciu, Victor Daniel Miron, Oana Săndulescu

**Affiliations:** 1 Carol Davila University of Medicine and Pharmacy, 050474 Bucharest, Romania; anca.simionescu@umfcd.ro (A.A.S.); anca.streinucercel@umfcd.ro (A.S.-C.); alexandrazotta@yahoo.com (A.M.A.S.); mariana.vieru@umfcd.ro (M.V.); mironvictordaniel@gmail.com (V.D.M.); oana.sandulescu@umfcd.ro (O.S.); 2 Department of Obstetrics and Gynecology, Filantropia Clinical Hospital, 011132 Bucharest, Romania; 3 National Institute for Infectious Diseases “Prof. Dr. Matei Balș”, 021105 Bucharest, Romania; 4 Department of Allergology and Clinical Immunology, Nicolae Malaxa Clinical Hospital, 022441 Bucharest, Romania; 5 National Institute for Mother and Child Health “Alessandrescu-Rusescu”, 127715 Bucharest, Romania; biamidan@yahoo.com

**Keywords:** pregnancy, vaccines, Europe

## Abstract

Vaccinations during pregnancy can protect the mother from several infections, thus blocking vertical transmission. Furthermore, through passive antibody transfer, the newborn can be protected against some infections in the first months of life until their own vaccination regimen is initiated and completed at the appropriate age. Pregnancy can be considered a high-risk condition that increases vulnerability to infectious diseases with potentially unfavorable evolution. We present the current knowledge on vaccination during pregnancy in Europe as a useful information source for different health workers involved in prenatal care. Many European countries implement vaccination policies specifically designed for pregnant women, but there is great heterogeneity among programs. Recommendations on vaccination during pregnancy must be based on current high-quality scientific data. The decisions must be made for each individual case, depending on the associated conditions or special circumstances, with a concomitant assessment of the potential benefits and risks to both the pregnant patient and the fetus. Many vaccines are well-tolerated in pregnant women, with no clinically meaningful injection site reactions, systemic symptoms, or vaccine-related serious adverse events.

## 1. Introduction

**Figure 1:**
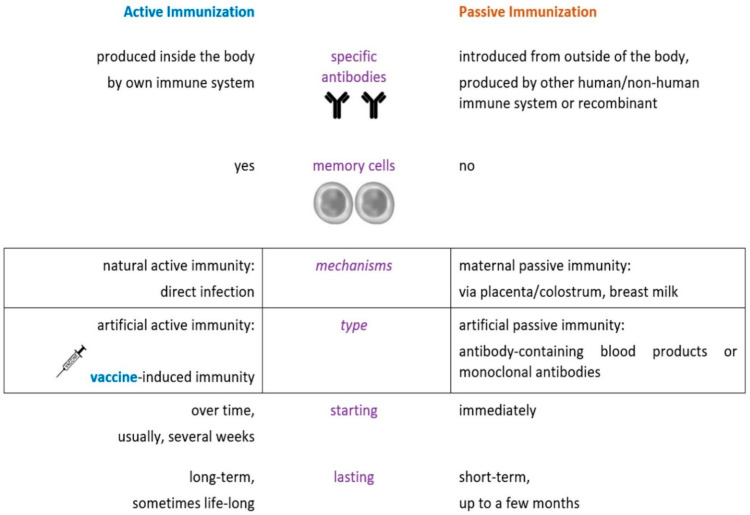
Position of vaccines as an active immunization strategy.

Vaccines are preparations that are used to stimulate the body’s immune response against pathogenic microorganisms; immunization is the process whereby a person acquires protection against a disease through vaccination, and vaccination is defined as the act of administering a vaccine to the body to induce protection from a specific disease, according to the most recent CDC (US Centers for Disease Control and Prevention) definitions of immunization terms [1]. The position of vaccines in immunization processes is presented in Figure 1 [2].

In the European Union (EU), the development of vaccines starts with preclinical in vitro and in vivo testing in the laboratory, after which they move into clinical testing, initially in phase 1, first-in-human clinical trials that are generally performed in healthy volunteers. After confirming their safety and pharmacokinetics in humans, vaccines are moved into phase 2 trials, which continue to assess their safety and pharmacokinetics in the targeted patient population and usually establish the dose that will be used for phase 3 trials, which are larger, pivotal clinical trials assessing the efficacy of the vaccine. Essentially, these clinical trials gather robust evidence on how the vaccines work and ensure that their benefits outweigh any potential side effects or risks. Once sufficient evidence has been gathered from research and clinical trials, the companies developing the vaccines can apply to the European Medicines Agency (EMA) for marketing authorization. The EMA evaluates all data by conducting independent and thorough scientific assessments, and based on the Agency’s scientific assessment, the European Commission decides whether to grant marketing authorization in the EU. The vaccine can then be used in clinical practice. However, the research process does not stop at this point. Pharmacovigilance activities start at the same time as the vaccine roll-out, and phase 4 postmarketing clinical trials continue to gather data on the safety and real-world effectiveness of the active immunization product [3].

The introduction of vaccinations has significantly changed the landscape of infectious diseases, transforming frequent communicable illnesses from major causes of morbidity, mortality, or debilitation into vaccine-preventable diseases. Many vaccines represent great success stories in public health and can be considered one of the major factors of sustainable development [3,4,5].

## 2. Overview of Vaccination during Pregnancy

Complex dynamic changes occur during pregnancy to avoid detrimental immune responses against the allogeneic fetus and to protect the mother and the fetus from pathogens. How these immunological adaptations contribute to the modulation of the risk of infection is still not clearly understood. Along with immune changes, other physiological factors must be considered, such as reduced functional residual lung capacity (due to increased abdominal pressure), with the risk of more severe pneumonia. Moreover, several infections, such as influenza, measles, mumps, and rubella, may be more frequently reported due to their severe adverse fetal outcomes [6].

Vaccination during pregnancy has a dual beneficial effect by preventing infection in the mother and the newborn/young infant. Pregnant women are at risk of vaccine-preventable disease-related morbidity and mortality, including adverse pregnancy outcomes. Influenza, tetanus, diphtheria, and pertussis are vaccine-preventable infectious diseases for which vaccinations are routinely recommended during pregnancy. By preventing infection in pregnant woman, vertical transmission is blocked. Moreover, passive immunity via maternal vaccine-induced transplacental antibody transfer affords protection to the newborn/young infant for up to six months of life. Only maternal IgG antibodies are transferred through the placenta (the IgG1 subclass is transferred most efficiently), facilitated by syncytiotrophoblast Fc receptors. This is especially important for influenza and pertussis, for which no active immunization options are available for infants [7,8].

All healthcare providers should routinely assess the vaccination status of their patients, particularly those who are pregnant or who wish to conceive [8]. Because the proportion of women who undergo vaccination or request information on vaccination options during or before pregnancy is not adequate for an epidemiological approach, the purpose of this narrative review was to summarize the current knowledge on vaccinations in pregnant women as a useful information source for health workers involved in pregnancy care. In addition, good-quality scientific information must be used to counteract myths and misconceptions regarding vaccinations during pregnancy, such as those stating that no prophylactic or therapeutic pharmaceutical products, including vaccines, should be administered to pregnant women.

The safety and effectiveness of vaccinations in pregnancy have long been a subject of debate, as most clinical trials specifically exclude pregnant persons, who are considered a “vulnerable” population [9]. In response to the claim that pregnant women cannot participate in clinical trials for ethical reasons, a different perspective is that their right to autonomy is violated by specifically excluding them from clinical trials with vaccines, thus depriving their personal right to protection against infectious diseases [10,11,12]. The inclusion of pregnant women in such trials should ensure enhanced safety monitoring and appropriate follow-up for mothers and their infants. If these criteria can be met, the benefits of active immunization can potentially extend to this patient population. “Pregnant women ought to be protected through research, not from research, in order to move forward in the promotion of health equity” [13].

The factors contributing to the reluctance or hesitancy of patients and/or medical staff with regard to vaccinations in pregnancy include: lack of proper information or limited patient information; poor health literacy; insufficient or inadequate training of the medical staff; lack of medical recommendations for vaccination; erroneous perception of the risks related to the infectious disease, including its potential severity and complications; fear of needles; distrust in the medical system and vaccinations; lack of vaccine availability; costs; and fake news, misinformation, and disinformation received from the media, relatives or friends [14]. Vaccine hesitancy, defined by the World Health Organization (WHO) as “a delay in acceptance or refusal of vaccination despite the availability of vaccination services,” is one of the top 10 threats to global health [15].

Many European countries implement particular vaccination policies designed for pregnant women, but there is a lack of homogeneity among vaccination programs in terms of recommended vaccines, as well as the timing of vaccinations [16]. Vaccination policies in European countries are influenced by differences in the incidence of vaccine-preventable diseases, vaccine uptake rates, reimbursement and cost issues, and the criteria used to establish the need to introduce vaccines in national vaccination programs [17]. More than half of European countries have implemented mandatory vaccinations for specific risk groups, such as healthcare personnel and/or children. However, no mandatory vaccination policy has been instated for pregnant women in Europe [16].

The decision to vaccinate should always weigh the risks and benefits of immunization on a case-by-case basis. Vaccination is recommended in cases in which the occurrence of the infection would pose a risk to the mother or the fetus, the mother is prone to acquiring the infection, an effective vaccine exists for that infection, maternal vaccination would bring a benefit to the mother or the fetus, or the benefits of the vaccination outweigh the risks [18,19].

At present, it is unanimously accepted that most inactivated vaccines can be administered to pregnant women without associated risks for the mother or the fetus [4,7]. Moreover, to date, no adverse pregnancy outcomes have been documented for COVID-19 vaccines, and this should be effectively communicated [8,15]. On the contrary, live-attenuated viral vaccines are not indicated during or immediately before pregnancy because of a theoretical concern about potential reversion to virulence and subsequent transmission to the fetus, although this risk appears to be low [7,8,13,14,15].

Vaccines approved in European countries for pregnant women are safe and effective [20]. Currently, according to their safety profiles and recommendations in vaccination programs or special circumstances and settings, vaccines administered during pregnancy in European countries may be classified (Table 1) as: routine vaccines; vaccines for special circumstances and settings with a high risk of exposure or severe disease if infection occurs (some of them with insufficient safety data or ongoing safety monitoring); contraindicated vaccines (live-attenuated viral vaccines); vaccines that may be used only in exceptional extenuating circumstances; and potential future vaccines in pregnancy.

## 3. Routine Vaccinations for Pregnant Women in European Countries

### 3.1. Influenza Vaccination

Inactivated influenza vaccines are routinely recommended in pregnancy. They have been almost universally accepted for influenza prophylaxis, since 1995–1996, when pregnant patients were recognized as a distinct risk group for influenza virus infection [21]. These inactivated flu vaccines can be safely administered at any time during pregnancy and are effective in preventing maternal influenza, which can be associated with major morbidity or serious cardiopulmonary complications [22].

A decline in natality and increased numbers of miscarriages were documented during the 1918 Spanish influenza pandemic, with first-trimester pregnancy losses occurring in 1 out of 10 pregnancies during the peak of the pandemic [23]. Influenza increases the risk of stillbirth. Moreover, during the H1N1 flu pandemic, multiple cases of fetal death, preterm birth, or low birth weight were reported [24]. During this 2009–2010 influenza pandemic, pregnant women had higher odds of being hospitalized or admitted to intensive care units than non-pregnant adults [25].

Influenza can occur in any trimester of pregnancy, with similar incidence rates [26]. During early pregnancy, it can be associated with obstetrical morbidity (premature birth, intrauterine growth restriction, and fetal distress), but also with unfavorable development in neonates [27,28,29]. Risks of cleft palate, neural tube defects, and heart malformations following influenza during pregnancy have been recognized, which are mainly associated with the occurrence of fever [30]. A high risk of either severe illness or complications is seen during the second and third trimesters. The highest risk occurs in the third trimester, with an increased risk of maternal respiratory complications. In this stage, the respiratory physiology is already altered by biochemical and hormonal changes and by mechanical factors, such as the distended pregnant uterus, which precludes complete diaphragmatic excursion [31,32,33,34]. The fetus and newborn benefit from the vaccination of a pregnant woman because of a lowered risk of stillbirth [35], premature birth, low birth weight, and neonatal death [36]. Influenza vaccination effectively lowers the rate of febrile respiratory illness in pregnant women [37]. Secondary health benefits can also be derived from preventing influenza. Data have linked fetal exposure to influenza virus, particularly when the infection occurs during the second or third trimester, with a subsequent increased risk of seizures during the first seven years of childhood [38]. Furthermore, by mounting an antibody response, transplacental antibody transfer also occurs, which ensures a certain degree of protection during the first months of life, reducing the risk of hospitalization due to influenza until the infant can effectively receive the influenza vaccine starting at six months of age [37,39].

A recent randomized study revealed that a quadrivalent inactivated influenza vaccine has similar immunogenicity to a trivalent inactivated one, and it is well-tolerated in pregnant women who are vaccinated during the second or third trimester. Furthermore, high titers of hemagglutination inhibition antibodies have been detected in the cord blood after delivery, meaning that maternal immunization is likely to confer protection to newborns via transplacental antibody transfer [40]. In recent years, the use of trivalent vaccines targeting two influenza A strains and one influenza B strain has shifted toward the use of quadrivalent influenza vaccines targeting two influenza A and two influenza B strains [41,42,43]. During pregnancy, quadrivalent vaccines are the preferred option [44]. All inactivated influenza vaccines are safe, immunogenic, and effective during pregnancy, as reported in randomized controlled trials, and no vaccine-related adverse events have been reported in newborns [40,44,45].

While there is a wealth of data confirming the safety and effectiveness of inactivated influenza vaccines during pregnancy, less information is available for other types of influenza vaccines. For recombinant influenza vaccines, more data regarding their use in pregnancy are still needed. Recently published recommendations from medical and public health experts state that women can and should be immunized during pregnancy with any licensed, recommended, and age-appropriate quadrivalent inactivated or recombinant influenza vaccine, but not with live-attenuated influenza vaccines. Influenza vaccines can be safely administered at any time point during pregnancy, whenever the influenza vaccination campaign starts in the region. Vaccination soon after the vaccine becomes available is also considered safe during the third trimester of pregnancy [46,47].

Most European countries have national recommendations for influenza vaccination during pregnancy: Albania, Austria, Belarus, Belgium, Croatia, Cyprus, the Czech Republic, Denmark, Estonia, Finland, France, Germany, Greece, Hungary, Iceland, Ireland, Italy, Latvia, Liechtenstein, Lithuania, Luxembourg, Malta, Monaco, the Netherlands, Norway, Poland, Portugal, Romania, Russia, Serbia, Slovenia, Spain, Sweden, Switzerland, Ukraine, and the United Kingdom. One dose of inactivated influenza vaccine is used in all countries. In Denmark, Germany, Malta, and Norway, influenza vaccination is systematically recommended in the second and third trimesters, while vaccination in the first trimester is recommended in the case of high-risk conditions only. In Sweden, vaccination is recommended starting from gestational week 16; while in the Netherlands, it is only recommended for high-risk pregnancy conditions, regardless of the trimester. In Austria, Portugal, and Russia, it is specified in the second or third gestational trimester; while Austria and Russia also recommend vaccination during the first trimester in epidemic seasons. In all other European countries, influenza vaccination is recommended for all pregnant women, regardless of trimester [16].

The World Health Organization (WHO) considers pregnant women a high-risk group that should be prioritized for influenza vaccination. Despite this, the rate of vaccination in pregnant women frequently remains low in some countries. For instance, in Romania, in the 2018–2019 period, only 0.1% of the doses (approximately 1300) offered free of charge by the Ministry of Health to influenza risk groups were administered to pregnant women [48]. This has to be improved, in line with the recommendations of the European Commission to increase influenza vaccine coverage in high-risk groups, among which pregnant women are included [16]. To conclude, pregnant women represent a particular risk group for influenza, in whom flu vaccinations should be prioritized and offered as part of routine medical care.

### 3.2. Tetanus, Diphtheria, and Pertussis Vaccination

The Tdap vaccine (the adult formulation of the tetanus, diphtheria, and pertussis vaccine) contains tetanus toxoid, diphtheria toxoid, and acellular pertussis antigens. Vaccination with Tdap is routinely recommended during pregnancy. Tdap booster vaccination during pregnancy is important for the prophylaxis of tetanus neonatorum, as well as diphtheria and whooping cough during a baby’s first months of life [8,16,17,18,19]. In the 1980s, the WHO established a vaccination initiative to eliminate maternal and neonatal tetanus, which markedly decreased mortality due to tetanus neonatorum [49,50].

Pregnant women who have never been fully vaccinated against tetanus and diphtheria should also receive a tetanus and diphtheria toxoid (Td) series. The recommendations regarding the timing of the booster dose vary from country to country. As a general underlying principle, a booster shot requires approximately two weeks to induce a sufficient antibody response, and antibody transfer through the placenta is most effective after gestational week 30 [48]. Although our review focuses on vaccinations in Europe, for a broader perspective, we consider it is important to mention that, in the United States, pregnant women are currently vaccinated with one booster dose of the Tdap vaccine in each pregnancy between 27 and 36 weeks of gestation, regardless of the time elapsed since the last Tdap booster dose [50,51]. This is carried out to maximize the maternal immune response and passive transfer of antibodies to the fetus [50,52,53]. High concentrations of diphtheria antitoxin, tetanus antitoxin, and antipertussis antibodies have been found in umbilical cord blood after Tdap administration during pregnancy [54]. For example, when Tdap was administered in the third trimester, antibodies transmitted to the fetus showed 77.7% efficacy against pertussis in newborns, which reached 90.5% against severe cases of pertussis; therefore, Tdap administration is an effective way to protect infants during the early months of life [55].

The vaccination strategy described is applicable to pregnant women who have previously been fully vaccinated against tetanus, diphtheria, and pertussis. If a woman has never received the primary vaccination schedule, it is recommended that three doses of a vaccine that contains tetanus and reduced diphtheria toxoid be administered: One baseline dose is followed by the second dose one month later and the third dose any time between 6 and 12 months after the second dose. It is recommended that Tdap should be chosen for one of the doses instead of Td, preferably for the one administered between 27 and 36 weeks of pregnancy [50,56]. Vaccination with Tdap is well-tolerated in pregnant women, with no clinically meaningful injection site reactions, systemic symptoms, or vaccine-related serious adverse events [57,58].

Many European countries have general recommendations for the vaccination of pregnant women against pertussis in the third trimester, widely defined as gestational weeks 27–36, or until labor under certain circumstances. Such countries include Austria, Cyprus, Greece, Italy, Poland, the Czech Republic, Denmark, Germany, Sweden, Ukraine, Serbia, Slovenia, Spain, Belgium, the United Kingdom, Estonia, Iceland, Luxembourg, Ireland, Portugal, and Romania. Certain countries only recommend vaccination under specific situations; for example, Moldova recommends vaccination in an epidemic context or in women with high-risk conditions, while Norway recommends it if local outbreaks occur. In particular settings, vaccination can be performed during the second trimester of pregnancy; for example, Denmark and Germany recommend it if premature labor is expected (however, not before gestational week 16 in Denmark), while other countries have a general recommendation for pertussis vaccination in the second (or third) trimester, such as in Liechtenstein, Switzerland, Belgium, the United Kingdom, Estonia, Iceland, Luxembourg, Ireland, Portugal, and Romania [16].

Importantly, while pertussis vaccines can be safely administered in any trimester of pregnancy, the epidemiological context should be taken into consideration when weighing the exact timing, depending on the main purpose of the vaccination: To protect the mother in an ongoing endemic or epidemic (if she has not previously received complete vaccination against pertussis) or to ensure the most efficient antibody transfer to the fetus, which generally occurs when the booster vaccination is administered toward the end of the second trimester or during the third trimester [59].

In the UK, the inactivated polio vaccine is administered as part of routine Tdap–IPV vaccination in pregnancy. Finland recommends pertussis vaccination in any pregnancy trimester (ideally close to the end of pregnancy). In France, the current recommendation is limited to the second or third trimester in residents of the Department of Mayotte, an overseas epidemic territory located in the northern Mozambique Channel in the Indian Ocean off the coast of Southeast Africa. In all of the abovementioned countries, the adult-type Tdap vaccine with or without the poliomyelitis component (Tdap–polio) can be used [16].

Among the European countries with active immunization regimens against pertussis during pregnancy, some also have targeted recommendations for tetanus vaccination. These are different depending on the prior immunization status of the mother. For example, in Belgium, in pregnant women who have not been vaccinated or have been incompletely vaccinated against tetanus, two doses of a tetanus-containing vaccine are recommended at any time during pregnancy, one of which should also contain acellular pertussis. This is to be followed up postpartum with a third vaccine dose. This general recommendation for unvaccinated or incompletely vaccinated women is also present in Finland and Estonia. In Spain, for pregnant women with a personal history of fewer than five doses of the tetanus vaccine, one to three doses of a tetanus-containing vaccine should be administered from weeks 27 to 36, one of which should contain a pertussis component (Tdap), preferably the one administered around weeks 27–28. In Moldova, unvaccinated pregnant women at risk of tetanus should receive one shot of a tetanus-containing vaccine in the third trimester. In Romania and Ukraine, vaccination with one tetanus-containing vaccine dose is recommended in the third trimester only if more than 10 years have elapsed from the last dose and/or no antibodies are detected. Countries such as Denmark and Germany have specific guidelines for postexposure tetanus vaccination in pregnant women, a medical procedure routinely performed in most countries for the management of tetanus-prone wounds, regardless of the existence of targeted pregnancy guidelines. In Norway, vaccination can be carried out if indicated, but ideally not during the first trimester if it can be safely postponed [16].

## 4. Vaccinations for Pregnant Women in Special Circumstances

### 4.1. Vaccination against COVID-19

Pregnancy is a risk factor for severe coronavirus disease 2019 (COVID-19), the new contagious disease caused by SARS-CoV-2 (severe acute respiratory syndrome coronavirus 2). COVID-19 vaccines represent one of the most important public health interventions taken to control the novel coronavirus pandemic. They provide individual benefits to vaccinees, particularly pregnant women, who are at risk of severe or critical COVID-19 outcomes, including death, and adverse pregnancy and neonatal outcomes, such as preterm birth, severe neonatal or perinatal morbidity, and mortality [60,61,62,63,64,65]. Although intrauterine transmission has been documented, it appears to be rare, which is hypothesized to be related to the low levels of SARS-CoV-2 viremia and the decreased co-expression of ACE2 (angiotensin-converting enzyme 2) and TMPRSS2 (transmembrane serine protease 2), which are needed for coronavirus entry into cells in the placenta [66]. SARS-CoV-2 infection during pregnancy has been associated with a number of adverse pregnancy outcomes, such as preeclampsia, preterm birth, and stillbirth. These associations during pregnancy have been reported mostly for severe COVID-19. Compared to asymptomatic infected subjects, severe COVID-19 illness during pregnancy increases the risk of cesarean delivery, postpartum hemorrhage, hypertensive gestational disorders, and preterm birth [60,67,68,69,70,71].

The initial clinical trials of COVID-19 vaccines specifically excluded pregnant women; therefore, limited safety data during pregnancy were available at the time of their emergency use authorization [72]. The currently available vaccines against COVID-19 in Europe can be administered to pregnant women. To date, none of the authorized COVID-19 vaccines use live-attenuated viruses, which are more likely to pose risks during pregnancy [73]. Although there is limited experience with the use of messenger RNA (mRNA) or adenoviral vector-based COVID-19 vaccines in pregnant women, animal studies have not reported direct or indirect harmful effects on the evolution of the pregnancy, embryo/fetal development, birth, or subsequent postnatal development. The European Medicines Agency will further review new information on these vaccines, and detailed information will be updated on its website as necessary [74,75,76,77]. For infections that occur during pregnancy more than two months prior to delivery, maternal SARS-CoV-2 IgG antibodies appear to be efficiently transferred across the placenta to the fetus and may persist for up to six months of life. Furthermore, for SARS-CoV-2 infections occurring perinatally, newborns have been shown to be capable of mounting strong antibody responses [78].

Safety data in pregnancy are rapidly being gathered from real-life settings. To date, no specific pregnancy-related risk signals have been reported. For any new vaccine, more information on pregnancy, birth, and newborn outcomes is needed, particularly if vaccine administration occurred earlier during pregnancy [66,67,72,79]. The risk of spontaneous abortion after mRNA COVID-19 vaccination does not appear to be higher than the baseline risk in the pregnant population. The odds of COVID-19 vaccine exposure among patients with spontaneous abortions were not increased in the prior 28 days compared to ongoing pregnancies [80]. The current WHO guidelines recommend the vaccination of pregnant women against COVID-19, particularly if they are at high risk of exposure or have comorbidities that enhance the risk of severe disease. Before getting vaccinated, pregnant women should discuss whether the benefits of vaccination outweigh the potential risks with their healthcare provider. The benefits may be greatest for those at the highest risk of COVID-19, such as frontline health workers, people residing in areas where high transmission occurs, and patients with comorbidities such as hypertension, obesity, or diabetes [73]. Moreover, early data from safety surveillance and pregnancy registries do not indicate particular safety signals regarding pregnancy or neonatal outcomes following vaccination against COVID-19 in the third trimester of pregnancy [81,82].

In addition to the fact that vaccination protects women against COVID-19 and its complications, preliminary data revealed that mRNA vaccination during pregnancy may induce neonatal protection against COVID-19 due to the transplacental transfer of SARS-CoV-2 antibodies. The mRNA vaccines induce a robust humoral immune response in both pregnant and lactating women. Moreover, the antibodies generated by the vaccine are detected in umbilical cord blood and breast milk [82,83,84,85,86,87]. The adverse event profile of COVID-19 vaccines appears to be similar in pregnant and non-pregnant women [88]. Importantly, despite many circulating fake news and conspiracy theories, there is no scientific evidence that any of the COVID-19 vaccines affect future fertility [88]. Findings showing that prenatal maternal COVID-19 vaccines have no adverse effects on pregnancy course or outcomes may help healthcare providers and pregnant women make informed decisions regarding vaccination against COVID-19 [89].

An increasing number of international professional societies and regulatory authorities, including the WHO, the European Medicines Agency, the UK Joint Committee on Vaccination and Immunization, and the US Centers for Disease Control and Prevention, either directly recommend COVID-19 vaccines or state that they may be considered during pregnancy in women at risk of exposure/infection and in those with any underlying comorbidities [90].

### 4.2. Hepatitis A Vaccination

This vaccine is not part of the schedule routinely recommended in pregnancy in many European countries, but it is indicated in women at high risk of hepatitis A virus (HAV) infection, because acute HAV infection in pregnancy is associated with high rates of gestational complications, such as meconium peritonitis, fetal ascites, polyhydramnios, and preterm delivery [91,92,93].

Theoretically, as an inactivated viral vaccine, it should be safe, and recent studies have confirmed its safety profile during pregnancy. There are three vaccines to protect against HAV on the market: two HAV vaccines, and one combined HAV and recombinant HBV vaccine; all three are inactivated vaccines and can be administered during pregnancy [94,95].

The hepatitis A vaccine can be administered to pregnant women at high risk of exposure or severe outcomes, such as during outbreaks; before traveling to underdeveloped countries where it is endemic; after close contact with persons having a high risk of hepatitis A infection, including those experiencing homelessness or using drugs or alcohol; in women receiving clotting factor concentrates; to those exposed to various biological specimens; or to those with occupational risk of HAV infection or a personal history of chronic liver disease or HIV [96,97,98,99].

Recommendations for the vaccination of pregnant women against hepatitis A are available on official government and national public health websites in only five European countries for specific groups or settings. In Spain, vaccination is administered if indicated, while in Italy and Norway, hepatitis A vaccination is recommended if the benefits outweigh the risks. In Estonia, pregnant women can also be actively immunized. In Greece, specific high-risk groups are defined for vaccinations during pregnancy. These include drug users, homeless people, women with chronic hepatic disease or HIV infection, or postexposure [16].

### 4.3. Hepatitis B Vaccination

Vertical transmission of the hepatitis B virus (HBV) is the main cause of HBV infection in children. If the virus is transmitted perinatally, the infection has a chronic evolution in most cases [100,101]. HBV vaccination during pregnancy may be indicated in pregnant women who are finalizing an immunization series started prior to conception and unvaccinated HBsAg-negative pregnant women who are at high risk of acquiring HBV. Those at high risk include: women who have sexual and household contact with an HBsAg-positive person; individuals engaged in drug-using practices (who have ever injected drugs) or high-risk sexual behaviors (individuals with multiple sexual partners or sexually transmitted diseases, transgender men who have unsafe sex with men); healthcare and public safety workers with exposure to blood or blood-contaminated body fluids; incarcerated persons; women with certain comorbid conditions (end-stage kidney disease, chronic liver disease, diabetes, hepatitis C virus, and/or HIV infection); and all unvaccinated individuals traveling to areas with an intermediate prevalence of HBV infection (the Mediterranean area, Eastern Europe, parts of Asia, and Latin and South America) to high (parts of sub-Saharan Africa) [102,103,104,105].

One of the two conventional recombinant HBV vaccines, or the combined HAV and recombinant HBV vaccine, may be recommended in pregnant women. A conventional recombinant HBV vaccine contains the hepatitis B surface antigen produced in yeast cells (*Saccharomyces cerevisiae*) by recombinant DNA technology, adsorbed on hydrated aluminum hydroxide, while the combined vaccine contains inactivated hepatitis A virus and recombinant hepatitis B surface antigen produced in the same yeast cells. When hepatitis B primary vaccination is indicated during pregnancy, the standard dosing intervals for adults should be used. No particular concerns regarding adverse events in pregnant women or their infants are mentioned. As a precautionary measure due to the limited amount of safety data, pregnant women are recommended to avoid the new recombinant HBV vaccine produced in yeast cells (*Hansenula polymorpha*) by recombinant DNA technology and comprising the hepatitis B surface antigen and an adjuvant represented by a short (22-mer) oligonucleotide sequence containing microbial DNA-like unmethylated CpG motifs [94,97,106].

Some European countries, such as Belgium, Greece, Norway, Serbia, and the United Kingdom, provide recommendations for hepatitis B vaccination in pregnant women at high risk of infection or severe disease, such as those with multiple sexual partners, intravenous drug users, and those with chronic hepatic disease. Germany, Denmark, and Finland recommend hepatitis B vaccination for postexposure prophylaxis, while Spain recommends it for pregnant women at risk of infection or postexposure. It is also indicated in Portugal, Estonia, and Italy if the benefits outweigh the risks [16].

### 4.4. Pneumococcal Vaccination

Because not enough data are available regarding vaccination during pregnancy, including on safety during the first trimester, in women with conditions that increase the risk of invasive pneumococcal disease, the 23-valent pneumococcal polysaccharide vaccine (PPSV23) should be received before conception. There are also insufficient data on the use of pneumococcal conjugate vaccines (PCV10 or PCV13) in pregnancy. Recent data revealed that PCV10 and PPSV23 are equally immunogenic and safe in pregnant women with HIV, conferring similar levels of protection to infants. To ensure the best antibody response, PPSV23 should follow in sequence the administration of a PCV vaccine by at least 8 weeks, ideally 1 year. In regions in which childhood PCV administration decreases the circulation of correspondent serotypes, PPSV23 may be more advantageous in HIV-infected pregnant women [107,108,109,110].

Recommendations for vaccination against pneumococcal disease during pregnancy are available in 10 European countries. Vaccination is recommended for pregnant women at high risk of pneumococcal disease or severe adverse outcomes (risk factors include chronic heart, lung, or liver disease, diabetes mellitus, alcoholism, cigarette smoking, functional or anatomic asplenia, or immunosuppression) in Greece; to high-risk groups during the second or third trimester in Spain; to women with asplenia in Sweden and Finland; during epidemics in Belgium; if indicated in Portugal and Estonia; if considered clearly necessary in Denmark; if vaccination cannot be delayed in Norway; and as an option after assessing benefits and risks in Germany [16].

### 4.5. Meningococcal Vaccination

In pregnant women at high risk of meningococcal disease, the benefits of vaccination are expected to outweigh potential risks. The quadrivalent meningococcal conjugate vaccines (MenACWY) seem safe for administration during pregnancy and may be used if otherwise indicated, while for the meningococcus serogroup B vaccines (MenB), decisions should be made after the risks and benefits are carefully assessed individually [106,111,112,113]. Recommendations for vaccination against meningococcal disease during pregnancy are issued in 10 European countries. This vaccination is administered to pregnant women with particular risk factors, such as complement deficiencies, HIV infection, or asplenia, in Greece; to similar high-risk groups or postexposure in Spain; and to women with asplenia or at risk of exposure in Finland. Meningococcal vaccination is administered in epidemics in Belgium and Norway and if its benefits outweigh the risks in Denmark, Germany, and Italy. Moreover, it may also be indicated in Portugal and Estonia [16].

### 4.6. Haemophilus Influenzae Type b Vaccination

The *Haemophilus influenzae* type b (Hib) conjugate vaccine is an inactivated vaccine recommended for pregnant women who did not receive the childhood Hib series and are at increased risk of invasive Hib disease due to certain chronic conditions, such as HIV infection, splenectomy, or sickle cell disease. In areas with a high incidence of invasive Hib disease in infants younger than six months, vaccination during pregnancy can reduce the risk of Hib disease in infants. Vaccination should be discussed on an individual basis. Published data suggest that Hib vaccination during the third trimester of pregnancy is immunogenic and safe [114,115]. Vaccination against *Haemophilus influenzae* can be considered for pregnant women with anatomical and functional asplenia in Finland. In Spain, it is recommended in the third trimester for pregnant women with chronic conditions if they were not vaccinated during childhood [16].

### 4.7. Poliomyelitis Vaccination

Poliomyelitis has been eradicated from most resource-rich countries, and vaccination against the disease is not routinely recommended in pregnant women but can be administered in cases of high risk of exposure. Pregnant women should be immunized if travel to certain regions where poliovirus transmission continues is unavoidable, such as Afghanistan, Pakistan, Madagascar, Yemen, and China [116]. Although two vaccines, the live-attenuated oral polio vaccine (OPV) and an inactivated polio vaccine (IPV), exist on the market, IPV is the only one recommended in pregnancy in persons at increased risk of infection according to the immunization schedule for adults. In the UK, it is routinely administered as part of the Tdap–IPV routine vaccination in pregnancy between gestational weeks 16 and 32. Adverse maternal and fetal effects of IPV administration have not been documented. Subsequent to mass immunization programs in response to poliovirus epidemics in Israel and Finland, when thousands of pregnant women were administered OPV, perinatal death, preterm birth, and congenital anomalies were not higher than expected [4,7,117,118]. According to current European vaccination programs, the recommendation for poliomyelitis vaccination of pregnant women exists in four European countries: In Germany for postexposure prophylaxis, in Portugal and Estonia if vaccination is indicated, and in Italy if the benefits outweigh the risks, but to be avoided in the first two months [16].

### 4.8. Rabies Vaccination

Rabies vaccination in pregnant women is permitted for pre-exposure prophylaxis if there is a high infection risk. The inactivated rabies vaccine can also be administered, with or without rabies immune globulin, for postexposure prophylaxis during pregnancy in many European countries. Rabies vaccination may also be used for pre- or postexposure prophylaxis in specific professional risk groups, such as veterinarians or speleologists. No increase in the risk of preterm birth, miscarriage, or fetal abnormalities has been observed after rabies vaccine exposure, and thus, unintended rabies vaccine exposure during pregnancy is not an indication for termination [119,120]. In Norway, pre-exposure rabies vaccination in pregnant women can be recommended after a careful benefit/risk evaluation, but not in the first trimester if a delay is reasonable. In France, Italy, Spain, Finland, and Russia, postexposure vaccination against rabies is permitted after a careful risk assessment. In Denmark, pregnant women can be vaccinated against rabies when the benefits to the mother are expected to outweigh the possible risks to the fetus/newborn child. In the United Kingdom, pregnancy is not considered a contraindication to postexposure prophylaxis due to the severity of this disease and, if there is a high risk of exposure to rabies, pre-exposure prophylaxis can also be recommended in pregnancy [16].

### 4.9. Tick-Borne Encephalitis Vaccination

Tick-borne encephalitis (TBE) is a central nervous system (CNS) flavivirus infection that can result in long-term neurological sequelae and even death. Very limited data are available regarding pregnancy outcomes for the mother and fetus if TBE infection occurs during pregnancy. The main risks are potentially related to complications occurring if severe disease develops in the mother. Inactivated whole-virus chicken embryonic fibroblast cell culture-derived TBE vaccines are available in Europe [121,122,123]. European countries with increased risk of TBE include Austria, Slovenia, Slovakia, Hungary, the Czech Republic, the Baltic States, southern Germany, southern and eastern Sweden, and Russia [124]. If vaccination was not performed prior to pregnancy and the risk of TBE is high, vaccination can be considered during pregnancy under certain conditions. An inactivated vaccine for tick-borne encephalitis (TBE) may be administered in pregnant women in Estonia. In Finland, TBE vaccination should be considered in pregnant women engaged in outdoor activities and who lived in risk areas for more than one month, while in Denmark and Norway, the TBE vaccine can be administered if absolutely needed, only after a detailed risk/benefit assessment. In Italy and Germany, it can be recommended for pregnant persons in specific risk areas, while in the latter country, it should be avoided in the first trimester of pregnancy [16].

### 4.10. Typhoid Fever Vaccination

Although infection with *Salmonella typhi* is not usually associated with an increased risk of adverse pregnancy outcomes, in utero infection leading to pregnancy loss and preterm labor has been reported [125,126,127]. Not enough data are available regarding typhoid vaccination during pregnancy. If exposure cannot be otherwise avoided due to traveling to typhoid-affected regions, pregnant women can be immunized with the inactive capsular polysaccharide vaccine (Typhim Vi) if needed, but not with the oral live-attenuated typhoid vaccine (Ty21a) [128].

### 4.11. Human Papillomavirus Vaccination

Several inactivated human papillomavirus (HPV) vaccines are available (bivalent, quadrivalent, and nonavalent) for the nonpregnant population in Europe, with a recent shift toward prioritizing the use of the nonavalent vaccine. Although some studies found no differences in the prevalence of oncogenic HPV types in women before and after pregnancy, the interactions between HPV infection and pregnancy need to be further evaluated [129].

Administration of any HPV vaccine during pregnancy is not usually recommended because the safety data are considered insufficient, and this type of vaccine is not considered urgent in most cases. Thus, vaccination should be postponed until the completion of pregnancy. If someone becomes pregnant after initiating the HPV vaccination schedule, the remaining doses should be continued after pregnancy, but there is no reason to be alarmed or to terminate the pregnancy. Data from inadvertent use in pregnant women are increasingly available and reassuring, indicating neither malformative nor fetal/neonatal toxicity, similar to animal studies. Moreover, women who have been recently vaccinated against HPV do not need to delay conception [130,131,132,133,134]. Human papillomavirus vaccination can be administered during pregnancy in Germany after a risk/benefit assessment [16].

### 4.12. Anthrax Vaccination

In exceptional extenuating circumstances, the anthrax vaccine, which would otherwise be avoided in pregnancy, can be administered to pregnant women following direct exposure, but these are only administered in consultation with the authorities in charge of the supervision of such medicinal products [135,136].

## 5. Vaccinations to Be Avoided during Pregnancy

Live-attenuated vaccines (LAVs), such as measles, mumps, and rubella (MMR), varicella, live-attenuated influenza, or yellow fever, are generally contraindicated during pregnancy, although the risk of adverse pregnancy outcomes after maternal immunization with these vaccines appears to be small. A recently published systematic review on the safety of LAVs during pregnancy revealed that the available evidence is mainly derived from historical cohort vaccination against smallpox and observational studies on the oral polio vaccine and vaccines against rubella, yellow fever, and dengue fever. Except for the smallpox vaccine, adverse pregnancy outcomes with documented cases of fetal vaccinia, albeit rare, have been reported [13]; maternal immunization with other LAVs reveals no evidence of an impact on pregnancy outcomes, but the existing evidence is of very poor quality. Pregnancy should be avoided for at least 28 days after administering a dose of the varicella vaccine [137] or a rubella-containing MMR vaccine [138]; this recommended time span has decreased in the last two decades from an initial recommendation of three months [139].

LAVs are currently contraindicated during pregnancy due to potential safety concerns for the mother and the fetus, particularly if administered during the first trimester of pregnancy. The hypothetical viral replication in the host is potentially capable of causing viremia with a risk of transplacental transmission to the developing fetus and potential adverse effects on pregnancy outcomes [140,141,142].

The measles, mumps, and rubella (MMR) LAV should not be given to pregnant women due to the theoretical risk of maternal morbidity and adverse outcomes associated with measles and rubella infections during pregnancy, coupled with the low risk of the LAV having the hypothetical potential to induce, at a very low rate, clinical or subclinical infection following vaccination. After inadvertent vaccination, the termination of pregnancy should not be recommended solely based on a theoretical risk of embryopathy [138,139]. Measles in late pregnancy can lead to perinatal infant infection, which may be associated with the risk of subacute sclerosing panencephalitis and high mortality. It is recommended to administer standard human immunoglobulin for postexposure prophylaxis in susceptible pregnant women who have been exposed to measles, with susceptibility being assessed by age and vaccination history and/or antibody testing [138,143].

Pregnant women without immunity to rubella should receive the MMR vaccine postpartum, even if breastfeeding. Seroconversion without serious infection has been reported in breastfeeding infants, although the rubella virus is excreted into breast milk [138,144]. Despite the clear teratogenic effect of primary infection with the wild-type rubella virus during pregnancy, no cases of congenital rubella syndrome have been documented in large cohorts of women inadvertently vaccinated during pregnancy or before conception. The complete absence of such a risk cannot be confirmed, and the possibility of congenital rubella syndrome seems to be less than 1 per 1000 exposed pregnancies [13].

The varicella vaccines should also not be recommended to pregnant women, and afterwards pregnancy should be avoided for 28 days following each dose. Congenital varicella syndrome appears in 1–2% of cases of maternal varicella infection [145]. Although a low risk of congenital varicella syndrome after vaccination cannot be ruled out, cases of this syndrome and increased prevalence of other birth defects have not been detected [137,145,146]. For postexposure prophylaxis in non-immune pregnant women, immediate varicella-zoster immune globulin is indicated, along with postpartum vaccination at least five months after the administration of immune globulin. Varicella DNA has not been detected in breast milk or infants after vaccination [147].

The live-attenuated influenza vaccine (LAIV) is contraindicated in pregnancy, although no adverse fetal outcomes or unusual pregnancy complications have been reported. In addition, pregnant persons do not need to avoid contact with those immunized with LAIV [148,149,150].

Since the yellow fever vaccine is an LAV, it poses a theoretical risk in pregnancy. Vaccination should be performed at least one month before pregnancy. However, contrary to other live vaccines, this LAV can be considered for administration during pregnancy if the risk of yellow fever virus exposure is high due to unavoidable travel to endemic regions of sub-Saharan Africa or tropical regions of South America. Moreover, unintended vaccination is not an indication for pregnancy termination. Fetal infection after vaccination occurs at a low rate and does not appear to be associated with a high risk of major congenital anomalies [151,152,153,154].

The tetravalent dengue vaccine, which may be used in overseas European territories situated in tropical endemic areas, is also contraindicated in pregnancy, similar to most LAVs [155].

## 6. Potential Future Vaccinations in Pregnancy

In this section, we discuss potential future vaccines that are not yet approved even for the general population, much less for pregnant women.

Group B *Streptococcus* (GBS) are commensal bacteria colonizing approximately 20–40% of women’s lower urogenital and gastrointestinal tracts [156]. Despite its commensal status, during pregnancy, it can be associated with maternal sepsis, preterm births, and stillbirths following ascending infection, and it is a known cause of neonatal pneumonia, sepsis, and meningitis. Many pregnant women colonized with GBS do not have sufficient antibody levels at delivery to provide passive protection to neonates and infants aged less than 90 days. The introduction of recommendations to screen for GBS carriage during late pregnancy and the prophylactic administration of antibiotics during labor has been shown to significantly reduce early-onset GBS disease but does not appear to have an effect on late-onset disease, occurring after the first week of life [157,158]. Neonatal vaccination is unlikely to be beneficial due to the rapid progress of early-onset GBS sepsis. There is ongoing interest in a vaccine against GBS in pregnancy, but the relatively low incidence of neonatal disease makes clinical trials difficult and the progress slow. Although a hexavalent Ia, Ib, II, III, IV, and V glycoconjugate vaccine has been developed, there are no commercially available GBS vaccines [157,159,160,161].

Respiratory syncytial virus (RSV) is an orthopneumovirus representing a leading cause of lower respiratory tract disease and hospitalization among infants. Several maternal vaccines are currently undergoing clinical development. A recombinant adjuvant RSV nanoparticle vaccine (RSV F vaccine) has been evaluated in pregnant women and was found to be safe, and it was not associated with major side effects [162,163,164,165].

Cytomegalovirus (CMV), a member of the Herpesviridae family, is among the most common congenitally transmitted pathogens. Congenital CMV infection is a major cause of progressive and developmental cognitive, hearing, and motor impairment in newborns. CMV immunology and the consequences of acute infection, latent infection, reactivation, and/or reinfection are challenging for the development of vaccines, but the highest risk to newborn appears to occur if the mother develops CMV primoinfection during pregnancy [165,166,167,168].

Ebola viruses (EBVs), in the Filoviridae family, are part of the group of viruses responsible for hemorrhagic fever, mainly in Africa. They are highly contagious, and the disease has a high mortality. Ebola disease occurring during pregnancy has been associated with an increased rate of poor outcomes in neonates [169]. Recommendations on vaccination against the Ebola virus in 2019 suggested recombinant vesicular stomatitis virus–Ebola vaccination in pregnant women with ongoing safety monitoring [170,171]. Very few cases of Ebola have been reported in Europe, with at least one case in each of Italy, Spain, and the UK imported from African regions with widespread viral transmission [172].

In the past several years, Zika virus infection has emerged as a major cause of fetal microcephaly and other congenital impacts, especially when this flavivirus infection occurs in early pregnancy [173,174]. Zika virus has had a minimal impact in Europe and has been limited to travelers returning from endemic regions with mosquito-borne transmission—a few sporadic cases have been identified, locally acquired through sexual transmission, and, for the first time in 2019, three autochthonous vector-borne transmissions [175]. Vaccine development against Zika has been accelerated to address concerns related to the occurrence of congenital effects if infection occurs during pregnancy. Almost 40 vaccines have been designed on different platforms, such as inactivated whole virus vaccines protein subunit vaccines, adenovirus-vectored vaccines, and DNA and mRNA vaccines, but none of them are yet commercially available [165,176,177].

Malaria is caused by parasites in the genus *Plasmodium* and is transmitted by female *Anopheles* mosquitos, and it is associated with a 3–4-fold increase in the risk of miscarriage and an increased risk of stillbirth. Malaria was endemic in Europe until the 1970s. In the last decade, almost all (99%) of the malaria cases reported each year in the European Union have been travel-related. Malaria transmission occurs in large areas of Africa, Asia, Central and South America, and the South Pacific. Several cases of local malaria transmission in Europe have been reported in Greece and northern part of Cyprus (*Plasmodium vivax*) and in France (*P. falciparum*). Actual treatment and prophylactic strategies reduce its detrimental effects on pregnancy outcomes, but do not eliminate them [178,179]. The adjuvanted RTS, S vaccine, containing a portion of *P. falciparum* circumsporozoite protein fused with hepatitis B surface antigen, is not approved for administration to pregnant women or to those of childbearing potential [180,181]. Research on other malaria vaccines in pregnancy is underway. The placental-binding parasites express a protein that binds to chondroitin sulfate A (CSA), called VAR2CSA, with regions of this complex protein serving as targets for the candidate PRIMVAC and PAMVAC vaccines (PRIMVAC targets the CSA-binding VAR2CSA protein from *P*. *falciparum,* and the PAMVAC also targets a recombinant fragment of VAR2CSA from *P. falciparum*) [182,183,184]. Sequestration of *P. falciparum*-infected erythrocytes expressing the VAR2CSA antigen in the placenta can lead to poor pregnancy outcomes, such as low birth weight and maternal anemia. Following administration of the PAMVAC candidate vaccine, anti-VAR2CSA IgG subclass responses were assessed in pregnant Beninese women. The associations between IgG3 subclass antibody levels and the risk of low birth weight, and between IgG4 and placental infections at delivery suggest that the protective mechanisms potentially through antibody responses against VAR2CSA rely on different IgG subclasses [185].

## 7. Conclusions

Obstetricians and other obstetric care providers should assess the vaccination status of their patients on a routine basis. The inclusion of pregnant women in clinical vaccine trials, with enhanced safety monitoring and appropriate follow-up, can potentially expand the benefits of immunization to this particular patient population. A comprehensive overview of vaccination policies for pregnant women in Europe is important for the obstetrician, who needs to be informed about national vaccination programs and international recommendations, including the number and type of recommended vaccines, their indications, and the timing of doses in the vaccination schedule. Their recommendation on vaccination during pregnancy must be based on current high-quality scientific data, and decisions must be made for each individual case, depending on the particularities of the woman and the pregnancy, particular associated conditions, or special circumstances, with a concomitant assessment of the potential benefits and risks to both the pregnant patient and the fetus.

Beyond vaccine recommendations, the effectiveness of vaccination policies implemented in real-life settings is based on vaccine uptake. Pregnant women must have easy access to vaccinations during their routine healthcare visits. National vaccination policies specific to pregnancy should be strengthened throughout Europe to offer protection against vaccine-preventable diseases to pregnant women and their offspring.

## Figures and Tables

**Table 1:** Classification of vaccines during pregnancy according to their safety profiles and recommendations in vaccination programs or special circumstances and settings.

Category of Vaccination during Pregnancy	Types and Comments	Active Immunization Products	Abbrv.
Routine vaccinations	Inactivated vaccines approved in European countries	Inactivated influenza vaccines	IIV3, IIV4
		Tetanus, diphtheria, pertussis vaccine	Tdap
Vaccinations in special circumstances and settings	Different non-LAV types of vaccines, some with ongoing safety monitoring	COVID-19 vaccine	
		Hepatitis A and B vaccines	HepA, HepB
		Pneumococcal vaccines	PPSV23, PCV13
		Meningococcal vaccines	MenACWY
		*Haemophilus influenzae* type b vaccine	Hib
		Inactivated polio vaccine	IPV
		Inactivated rabies vaccine	RAB
		Inactivated tick encephalitis vaccine	TBE
		Inactive typhoid vaccine	Ty21a
		Human papillomavirus vaccine *	HPV
Contraindicated vaccinations	LAV vaccines contraindicated during pregnancy	Measles, mumps, and rubella vaccine	MMR
		Varicella vaccine	VAR
		Live-attenuated influenza vaccine	LAIV
		Live zoster (shingles) vaccine	ZVL
		Yellow fever and dengue vaccines	YF, DEN

Legend: Abbrv. = abbreviation; * HPV vaccine is generally not recommended during pregnancy due to a lack of data regarding its safety and efficacy in this population; IIV3, IIV4 = inactivated influenza vaccines trivalent, tetravalent; Tdap = tetanus toxoid, reduced diphtheria toxoid, and acellular pertussis; COVID-19 = coronavirus disease 2019; Hib = *Haemophilus influenzae* type b vaccine; HPV = human papillomavirus; MMR = measles, mumps, and rubella; LAV = live-attenuated virus.

## Data Availability

Not applicable.
